# Novel Technologies for Gastrointestinal Cancer Detection: A Systematic Review and Meta-Analysis of Diagnostic Accuracy

**DOI:** 10.14309/ctg.0000000000001058

**Published:** 2026-06-04

**Authors:** Melissa Barlow, Sarah Bailey, Saleh Alessy, Badr Al-Bawardy, Sadeem Aldarweesh, Rowaedh A. Bawaked, Heba Alharith, Amin Mahdi, Asrar Alaklabi, Mohammad Mawardi, Aldanah M. Althwanay, Nora A. Althumiri, Saleh Alabbad, Khalid I. Bzeizi, Severin Rakic, Ahmed Allehebi, Predrag Zivotic, Volkan Cetinkaya, Bandar AlJudaibi, Saleh A. Alqahtani

**Affiliations:** 1 Department of Health and Community Sciences, University of Exeter, Exeter, UK;; 2 Public Health Department, College of Health Sciences, Saudi Electronic University, Riyadh, Saudi Arabia;; 3 Liver, Digestive, & Lifestyle Health Research Section, King Faisal Specialist Hospital & Research Center, Riyadh, Saudi Arabia;; 4 Faculty of Life Sciences and Medicine, Centre for Cancer, Society and Public Health, King's College London, London, UK;; 5 College of Medicine, Al Faisal University, Riyadh, Saudi Arabia;; 6 Department of Internal Medicine, Division of Gastroenterology and Hepatology, King Faisal Specialist Hospital and Research Center, Riyadh, Saudi Arabia;; 7 Department of Internal Medicine, Section of Digestive Diseases, Yale School of Medicine, New Haven, Connecticut, USA;; 8 Al Salama Hospital, Jeddah, Saudi Arabia;; 9 Department of Medicine, Gastroenterology Section, King Faisal Specialist Hospital & Research Center, Jeddah, Saudi Arabia;; 10 Division of Gastroenterology, Washington University in St. Louis, St. Louis, Missouri, USA;; 11 Informed Decision Making, Riyadh, Saudi Arabia;; 12 Organ Transplant Center of Excellence, King Faisal Specialist Hospital & Research Center, Riyadh, Saudi Arabia;; 13 World Bank Group, Washington, District of Columbia, USA;; 14 Department of Medical Oncology, King Faisal Specialist Hospital & Research Center, Jeddah, Saudi Arabia;; 15 Division of Gastroenterology and Hepatology, Weill Cornell Medicine, New York, New York, USA.

**Keywords:** gastrointestinal neoplasms, early cancer detection, diagnostic accuracy, cancer screening, biomarkers, artificial intelligence, fecal immunochemical test, systematic review and meta-analysis

## Abstract

**INTRODUCTION:**

Gastrointestinal (GI) cancers account for a quarter of all cancers and one-third of cancer-related deaths worldwide. Novel diagnostic approaches are crucial to enhance early detection and improve patient outcomes. This review systematically identified and evaluated novel GI cancer detection approaches.

**METHODS:**

A systematic review was conducted using PubMed, EMBASE, Scopus, and the Cochrane Library on December 11, 2024, and updated on June 2, 2025. Cohort studies and randomized controlled studies, published in English, that evaluated a novel diagnostic approach for incident GI cancer in a clinical setting, included ≥300 participants, and reported diagnostic accuracy metrics were eligible for inclusion. Two reviewers independently screened studies and assessed risk of bias using the Quality Assessment of Diagnostic Accuracy Studies 2 tool.

**RESULTS:**

Of 9,604 records screened, 72 studies were included: by site, 51 on colorectal, 1 on upper GI, 1 on esophageal, 7 on gastric, 9 on pancreatic, and 5 on hepatocellular carcinoma. Stool DNA testing in patients with a positive fecal immunochemical test showed the highest performance for colorectal cancer (area under the receiver operating characteristic curve [AUC] 0.94–0.98). The best performing technologies across other cancer types were Q-Cancer for pancreatic (AUC 0.89–0.92), the GALADUS model for hepatocellular carcinoma in cirrhotic patients (AUC 0.94), and an artificial intelligence model for esophageal cancer (AUC 0.87).

**DISCUSSION:**

This review highlights several promising novel diagnostic approaches for GI cancer detection. External validation, population-level studies, and health economic evaluation are crucial to establish clinical utility across different settings and populations. Clinicians and policy makers must weigh the added value of novel technologies against current practice, regulatory requirements, clinical understanding, interpretation of results, and public trust.

## INTRODUCTION

Gastrointestinal (GI) malignancies represent a significant global health burden regarding morbidity and mortality, with around 4.8 million new cases and 3.4 million deaths worldwide each year (1). Early-stage diagnosis across all GI cancers is vital for improving survival outcomes. For example, the 1-year survival rate for stage I colon cancer is 97.6%, compared with 40.7% for stage IV (2). Curative treatment options, such as endoscopic resection or surgery, are typically available for early-stage cancers but are limited in advanced disease, when tumors are more likely to be unresectable, treatments are less effective, and metastatic spread significantly reduces the chances of survival.

Given the substantial health burden of GI cancers and the proven benefits of earlier diagnosis on stage at presentation and survival, novel approaches are needed to expedite diagnosis and reduce morbidity and mortality (3,4). Novel technologies for cancer detection, including blood-based and stool-based biomarkers and artificial intelligence (AI)-based predictive algorithms, purport to offer improved diagnostic accuracy at an early disease stage with noninvasive approaches (5,6). These technologies can be applied at several stages along the diagnostic pathway, from screening programs (targeting asymptomatic individuals) to primary care (for patients consulting a general practitioner with symptoms), and secondary care (where the diagnoses are typically confirmed). Despite the growing number of published technologies for earlier GI cancer detection, very few have made their way into routine clinical practice (6).

There is a need for a comprehensive review to synthesize the current landscape of novel technologies for GI cancer diagnosis, specifically those not already in routine clinical use, and to identify those with the greatest potential for clinical utility and successful real-world implementation. This systematic review aimed to address that gap by informing policy makers, clinicians, and researchers about novel approaches supported by high-quality evidence for diagnostic performance.

## METHODS

This systematic review was conducted in accordance with the Preferred Reporting Items for Systematic Reviews and Meta-Analyses (PRISMA) reporting guidelines. A study protocol was registered on the International Prospective Register of Systematic Reviews (PROSPERO) database (CRD42024625512) on 10th December 2024, before the commencement of title and abstract screening (7).

### Search strategy

PubMed, EMBASE, Scopus, and the Cochrane Library were searched for studies published from January 1, 2005, to December 11, 2024. An updated search was conducted to capture studies published from December 11, 2024, to June 2, 2025. The search strategy is provided in Supplementary Materials 1 (http://links.lww.com/CTG/B538).

Search results were uploaded into Covidence (Veritas Health Innovation, Melbourne, Australia), and duplicates were removed. Two reviewers independently screened titles, abstracts, and full texts for eligibility, and conflicts were resolved through discussion, or consultation with a third reviewer, when necessary. Interrater reliability was assessed using the Cohen kappa. Forward and backward citation chasing was performed to identify additional relevant studies.

### Eligibility criteria

A critical component of this study was to review novel diagnostic tests and technologies that had been studied in adult populations likely to accurately reflect the target clinical group. For this reason, only cohort studies and randomized controlled trials conducted in specific healthcare settings or patient populations were eligible for inclusion. Novel was defined as tests or technologies not well established in routine care, and/or new combinations of existing tests, and/or established tests using alternative thresholds or testing strategies. At least 300 participants per study was required, in accordance with previous literature (8). Case-control studies and studies that were conducted on nonspecific healthcare populations were excluded, as were studies published over 20 years ago (before 2005). Included tests and technologies were those not currently in routine clinical use. The full inclusion and exclusion criteria are outlined in Supplementary Materials 2 (http://links.lww.com/CTG/B538).

### Data extraction

One reviewer extracted relevant data from each study using a predefined data extraction form. A second reviewer independently verified extracted data for discrepancies. For each cancer site and for each test or technology assessed, we extracted: healthcare setting and patient populations (including country, and age and sex distributions), reference standard, threshold used to define a positive result, and the diagnostic performance of the test (sensitivity, specificity, positive predictive value [PPV], negative predictive value [NPV], and area under the receiver operating characteristic [ROC] curve [AUC]). The sensitivity of a test indicates the percentage of patients with the disease in question who test positive at a given threshold. Specificity indicates the percentage of true negatives among those without the disease. The PPV and NPV are the percentages of patients with a positive or negative test, respectively, who do or do not have the disease in question. The AUC is an overall indicator of diagnostic test performance derived from sensitivity and specificity. For clinical prediction models and AI models, data were extracted from external validation cohorts.

### Risk of bias assessment

Two reviewers independently assessed the risk of bias of each included study using the Quality Assessment of Diagnostic Accuracy Studies 2 (QUADAS-2) tool (9). Discrepancies were resolved through discussion.

For prediction models that reported diagnostic performance metrics only for the development cohort, the risk of bias was appropriately reflected in the concerns regarding applicability for patient selection.

### Data synthesis

Data were synthesized narratively unless 3 or more studies reported the diagnostic performance of the same test within a comparable healthcare setting, in which case a meta-analysis was conducted using Stata version 18.5 (10). A bivariate random-effects model was applied to pool diagnostic sensitivity and specificity using the *metadta *command (11). Between-study variance was assessed using τ^2^, heterogeneity was reported using I^2^, and the correlation between sensitivity and specificity across studies was summarized by ρ. Results were visually presented using forest plots and summary ROC (SROC) curves. Sensitivity analyses were performed to explore potential sources of heterogeneity. For the narrative synthesis, 95% confidence intervals (CIs) were reported when available in the original publications.

## RESULTS

**Figure 1.**
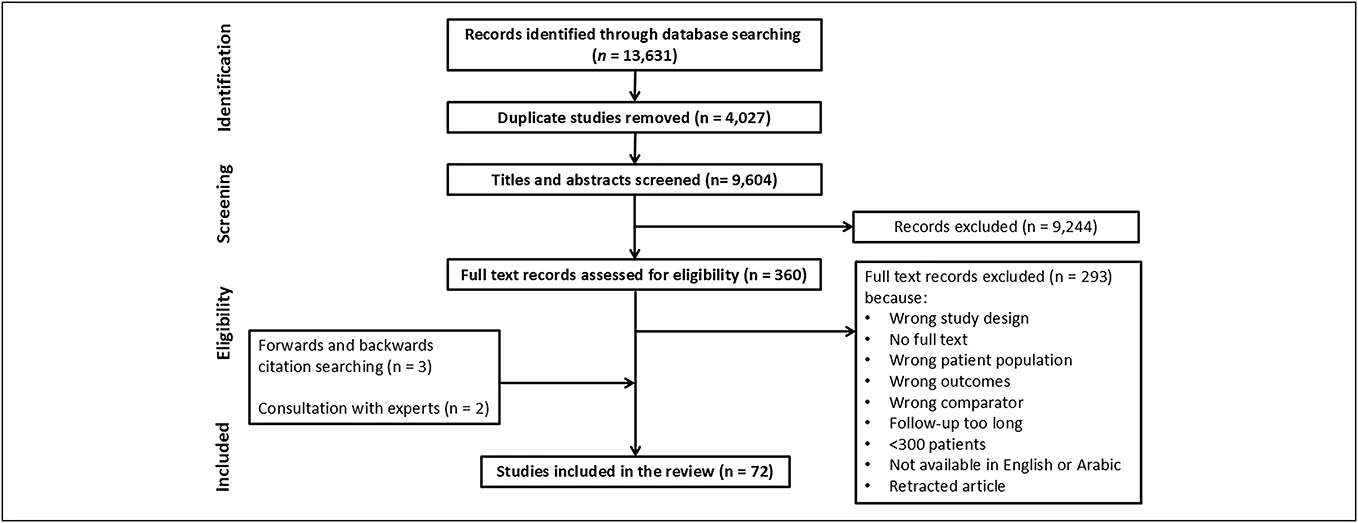
PRISMA flow diagram of the study selection process.

The search returned 13,631 records, of which 4,027 were duplicates. Titles and abstracts of 9,604 studies were screened for eligibility, and 9,244 were excluded. The full texts of 360 studies were reviewed; 293 were ineligible. Two further studies were identified through expert consultation, and 3 through forward and backward citation searching, resulting in 72 studies eligible for inclusion (Figure 1). Interrater reliability ranged from 0.32 to 0.88 for title and abstract screening, and from 0.64 to 0.97 for full-text screening.

The 72 eligible studies reported the diagnostic performance of 64 tests or technologies for GI cancer detection from 16 countries: 51 studies for colorectal cancer (CRC), 1 on upper GI, 1 on esophageal, 7 on gastric, and 9 on pancreatic and 5 on hepatocellular carcinoma (HCC). A summary of the included studies, including study-level data on patient numbers, study country, reference standard for cancer diagnosis, positive test threshold, and diagnostic performance metrics, is provided in Supplementary Materials 3 (http://links.lww.com/CTG/B538), stratified by the clinical setting in which the test or technology was evaluated (e.g. asymptomatic, primary care, or secondary care). Supplementary Materials 4 (http://links.lww.com/CTG/B539) includes the full data extraction table including additional data on test brand, sex and age distributions, clinical prediction model variables, and follow-up duration. The results of the QUADAS-2 risk of bias assessment were mixed across included studies (Supplementary Materials 5, http://links.lww.com/CTG/B540); no studies were excluded on the basis of risk of bias.

### Colorectal cancer

Of the 51 CRC studies, 25 evaluated the diagnostic accuracy of fecal immunochemical test (FIT). The reference standard for CRC diagnosis in these studies included colonoscopy, biopsy, and medical records. Although FIT is well established and widely implemented, these studies evaluated different applications of its use, including various thresholds and multiple tests. For comparability, these are summarized below by healthcare setting.

#### FIT in asymptomatic populations.

**Figure 2.**
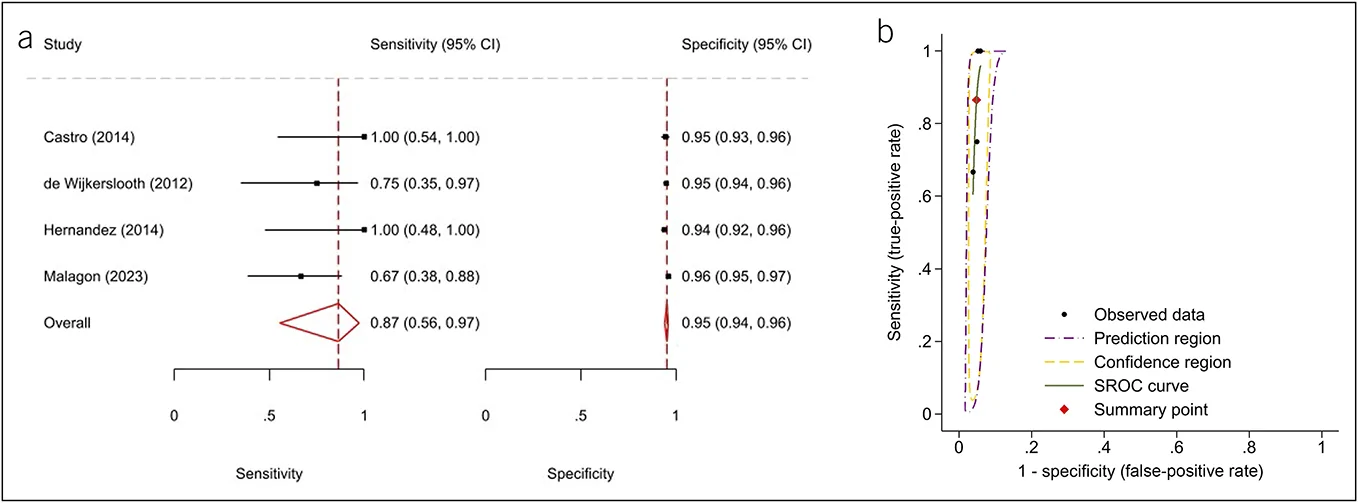
(**a**) Forest plot of pooled sensitivity and specificity of FIT for colorectal cancer detection at a threshold of ≥20 µg Hb/g in asymptomatic/screening populations. (**b**) Summary receiver operating characteristic plot of the diagnostic accuracy of FIT for colorectal cancer detection at a threshold of ≥20 µg Hb/g in asymptomatic/screening populations. CI, confidence interval; FIT, fecal immunochemical test; SROC, summary receiver operating characteristic.

Eight studies evaluated FIT in asymptomatic/screening populations with AUCs ranging from 0.97 to 0.76 (12–19). Six studies used a cutoff of ≥20 µg fecal hemoglobin (Hb)/g; 4 reported sufficient data for inclusion in a meta-analysis (12–14,16), where the pooled sensitivity of FIT was 87% (95% CI, 56 to 97) and specificity was 95% (94–96) (Figure 2a). There was a large region of prediction and confidence outlined in the SROC (Figure 2b). Between-study variance and heterogeneity were negligible (τ^2^ = 0.00, *I*^2^ = 0.02%) with an almost perfect negative correlation between sensitivity and specificity across studies (ρ = −1.000). However, there was substantial variance and heterogeneity in sensitivity (τ^2^ = 0.75, *I*^2^ = 31.8%) and moderate heterogeneity in specificity (τ^2^ = 0.02, *I*^2^ = 46.4%), indicating that FIT performance varied more in specificity than sensitivity across the included studies and populations. A sensitivity meta-analysis, excluding 1 study that recruited high-risk participants, yielded similar results (Supplementary Materials 6, http://links.lww.com/CTG/B538) (12).

There was little evidence of any meaningful differences in diagnostic performance at other thresholds, up to ≥40 µg Hb/g (12,14).

#### FIT in primary care.

**Figure 3.**
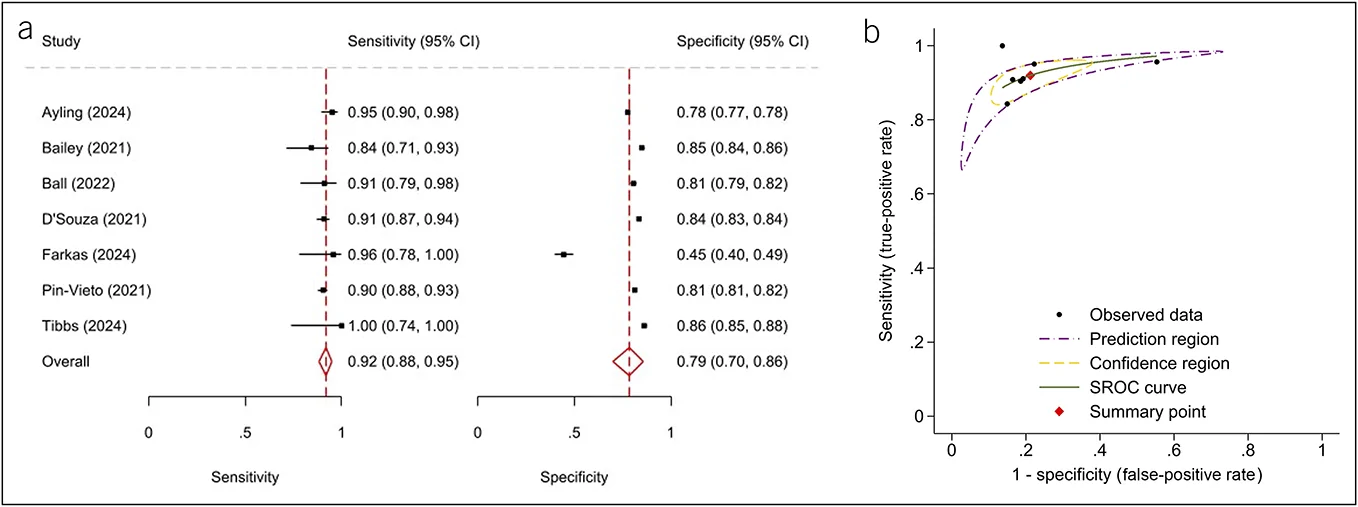
(**a**) Forest plot of pooled sensitivity and specificity of FIT for colorectal cancer detection at a threshold of ≥10 µg Hb/g in primary care. (**b**) Summary receiver operating characteristic plot of the diagnostic accuracy of FIT for colorectal cancer detection at a threshold of ≥10 µg Hb/g in primary care. CI, confidence interval; FIT, fecal immunochemical test; SROC, summary receiver operating characteristic.

Nine studies evaluated the diagnostic performance of FIT in primary care settings (symptomatic patients); all were assessed as having a low risk of bias, with AUCs ranging from 0.89 (95% CI, 0.82–0.95) to 0.93 (0.92–0.95) (20–28). Seven studies using a threshold of ≥10 µg Hb/g were pooled in a meta-analysis (20–22,24,25,27,28), with sensitivity and specificity of 92% (88–95) and 79% (70–86), respectively (Figure 3a). The regions of confidence and prediction in the SROC curve were small (Figure 3b). Between-study variance and heterogeneity were very low with an almost perfect negative correlation between sensitivity and specificity (*I*^2^ = 0.09%, τ^2^ = 0.00, ρ = −1.00). There were substantial heterogeneity and variance in specificity (*I*^2^ = 99.19%, τ^2^ = 0.41), but low-to-moderate levels of heterogeneity for sensitivity (*I*^2^ = 35.71, τ^2^ = 0.21). A sensitivity analysis excluding Farkas et al (2024) (25) reduced between-study variance (τ^2^ = 0.04 and 0.05 for specificity and sensitivity, respectively) but did not meaningfully improve heterogeneity for specificity (*I*^2^ = 96.74%) and yielded similar results (Supplementary Materials 7, http://links.lww.com/CTG/B538).

**Figure 4.**
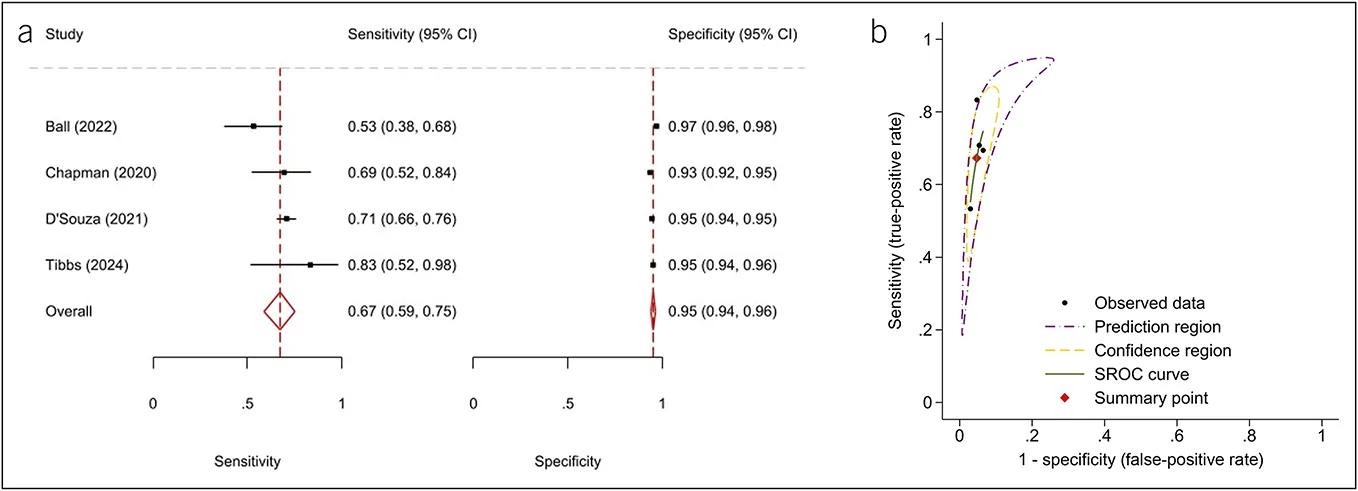
(**a**) Forest plot of pooled sensitivity and specificity of FIT for colorectal cancer detection at a threshold of ≥150 µg Hb/g in primary care. (**b**) Summary receiver operating characteristic plot of the diagnostic accuracy of FIT for colorectal cancer detection at a threshold of ≥150 µg Hb/g in primary care. CI, confidence interval; FIT, fecal immunochemical test; SROC, summary receiver operating characteristic.

Four studies with a threshold of ≥150 µg Hb/g in primary care were pooled in a meta-analysis (22–24,28) with a sensitivity of 67% (59–75) and specificity 95% (94–96) (Figure 4a, SROC plot Figure 4b). Between-study heterogeneity and variance were low overall (τ^2^ = 0.00, *I*^2^ = 0.09%, ρ = −1.00), with substantially higher heterogeneity for specificity (τ^2^ = 0.42, *I*^2^ = 99.2%) compared with sensitivity (τ^2^ = 0.21, *I*^2^ = 35.17%).

#### FIT in secondary care.

Seven studies evaluated FIT in secondary care settings among referred symptomatic patients (29–35); AUCs ranged from 0.86 to 0.89, and all had a relatively low risk of bias assessment. The thresholds used in each study to determine a positive result ranged from ≥7 to ≥19 µg Hb/g, and there was little evidence of variation in accuracy by threshold in this range.

#### Repeat FITs.

Among the studies of repeat FITs, 3 required 2 samples from included patients (12,32,34), and 2 required 3 FITs (25,30). The largest study of repeat FITs included 28,622 patients who completed 2 FITs (32); specificity was 81.6% (95% CI 81.1–82.0) when both FITs were positive, compared with 66.2% (95% CI 65.7–66.7) when either FIT was positive. The PPV of 2 positive FITs was 5.2%, compared with 3.1% when either FIT was positive. There was little clinically meaningful difference in sensitivity between the 2 approaches (91.5% for 2 positive FITs vs 97.8% when either FIT is positive). The time interval between tests varied from consecutive days (30) up to 1 week (12).

#### Other tests for colorectal cancer.

Thirty-two additional tests for CRC were evaluated. One study of FIT in combination with flexible sigmoidoscopy in an asymptomatic population reported an extremely high AUC of 0.99 (0.99–1.00), although this was not superior to FIT alone in the same study (0.96 (0.91–0.99)) (17).

Among the stool-based tests, 8 studies were in asymptomatic populations (16,18,19,36–39) and 4 in secondary care settings (30,34,40,41). Excellent diagnostic performance was observed in Cologuard 2.0 (19), which had a sensitivity of 93.9% (87.1–97.7) and specificity of 90.6% (90.1–91.0), although it was evaluated in patients already undergoing screening colonoscopy.

Three tests were blood-based (42–44). In secondary care, an evaluation of targeted methylation-based multicancer early detection (MCED) provided high specificity, PPV, and NPV (99.1%, 83.8%, and 97.8%, respectively) for CRC, with a low sensitivity of 68.5% (43).

Seventeen studies evaluated algorithm-based technologies for CRC detection; none of those in asymptomatic populations outperformed FIT alone (15,45–54). Of the 4 studies conducted in secondary care (55–58), COLONPREDICT and FAST reported high AUCs of 0.92 (0.90–0.94) and 0.91 (0.90–0.93), respectively (55,56). Both included FIT in the models and were assessed as having a low risk of bias.

### Upper GI cancer

A targeted MCED evaluated in 1,021 urgently referred patients in the UK identified 46 cases of upper GI cancer (43). The sensitivity of the MCED for any upper GI cancer was 80.4% (66.1–90.6), specificity was 98.1% (97.0–98.8), PPV was 66.1% (52.2–78.2), and NPV was 99.1% (98.2–99.6) (43). Diagnosis was established by upper GI endoscopy. Only 11 of the 46 cases (24.0%) were identified at an early stage.

### Esophageal cancer

One study evaluating esophageal cancer detection in secondary care was identified (59), which developed and internally validated a logistic regression model and an artificial neural network model based on multiple clinical factors. The AI model demonstrated slightly superior performance, with an AUC of 0.87 (0.83–0.89) compared with 0.82 (0.78–0.86) for the logistic regression model (59). The reference standard for diagnosis was upper endoscopy, and this study was assessed as having a low risk of bias.

### Gastric cancer

Seven studies evaluated 9 technologies for gastric cancer detection, with mixed results in the quality assessment (60–66). Reference standard diagnoses included endoscopy with biopsy or confirmation from medical records. Serum pepsinogen (PG) I and II had limited of sensitivity 67% (63–71) and specificity of 47% (43–51) in a screening population (63). Cai et al (2019)'s (60) clinical prediction model included PG I and II plus age, sex, gastrin-17 (G-17) level, anti-*Helicobacter pylori* (anti-Hp) immunoglobulin G antibodies, achieving a sensitivity of 70.7% (65.5–76.1) and specificity of 67.8% (66.9–68.8). Both studies were assessed as having a low risk of bias.

In secondary care, Zhou et al (2022)'s scoring system, incorporating age, sex, PG I/II ratio, G-17, and HP-IgG antibody, was outperformed by a 12-miRNA panel (AUCs 0.79 and 0.85 [0.81–0.88], respectively) developed by So et al (2021) (66); both studies were assessed as having a high risk of bias in more than 1 domain.

### Pancreatic cancer

There were 9 studies of prediction models for the detection of pancreatic cancer using electronic health record data from primary and secondary care settings (67–75) and cancer registries or medical records to confirm diagnosis. There was a high or mixed risk of bias across these studies.

The “QCancer” algorithm (including smoking status, diabetes status, chronic pancreatitis status, and a selection of recent symptoms) (72) had an AUC of 0.89 for men and 0.92 for women in an independent external validation including 2,150,322 primary care patients, with a low risk of bias (71).

For patients with preexisting health conditions, the Enriching New-Onset Diabetes for Pancreatic Cancer Model (73) had an AUC of 0.75 in an external validation (69). Boursi et al's (67,68) clinical prediction model for patients with new-onset diabetes had an AUC of 0.82 (0.75–0.89), with a low risk of bias and little evidence of overfitting, indicating that performance is likely to be similar when the models are applied to external data.

### Hepatocellular carcinoma

Four of the 5 included HCC studies evaluated the performance of diagnostic tests and technologies for patients at high risk of HCC with existing liver conditions (hepatitis B, hepatitis C, and cirrhosis) (76–79). HCC diagnosis was confirmed using a range of methods including medical record confirmation and established guidelines.

The best performing technologies incorporated alpha-fetoprotein and ultrasonography, which are widely accepted as the standard-of-care approach for HCC surveillance in high-risk patients. The gender, age, alpha-fetoprotein-L3, alpha-fetoprotein, des-gamma-carboxyprothrombin, and ultrasonography (GALADUS) model, with an AUC of 0.94 (0.92–0.94), significantly outperformed the standard approach of ultrasonography and alpha-fetoprotein (AUC 0.84, 0.82 to 0.85), with a notable increase in sensitivity (91.0% vs 70.8%, respectively) (79). However, due to the absence of external validation, this study raised applicability concerns regarding patient selection (Supplementary Materials 5, http://links.lww.com/CTG/B540).

## DISCUSSION

### Summary of findings

This review identified several technologies which show promise for real-world implementation of clinical effectiveness, which policy makers may want to consider for application either as new approaches or as modifications to existing early diagnosis strategies. QCancer (pancreatic), Cologuard (CRC screening), COLONPREDICT, and FAST (patients referred for suspected CRC) had the best diagnostic performance in studies at low risk of bias.

The most extensively studied technology, FIT, is widely used for CRC detection, and our meta-analysis supports its use in all healthcare settings, albeit with varying thresholds that will be influenced by local capacity and priorities as much as by optimal diagnostic accuracy. This review highlights the potential of stool-based DNA tests to improve the accuracy of FIT, particularly in asymptomatic populations. However, algorithm-based tests did not improve the diagnostic accuracy of FIT alone in screening or primary care settings. In secondary care, algorithms including FIT did demonstrate superior diagnostic accuracy.

Only 1 MCED test meeting the eligibility criteria was included in this review, conducted in secondary care settings in the UK (43). Although the diagnostic performance metrics for both upper and lower GI cancers were reasonable (albeit with wide CIs), the percentage of upper GI cancers identified at an early stage by the MCED (24.0%) was comparable with current early diagnosis rates in the UK for stomach (27.4%), pancreatic (22.6%), and esophagogastric cancers (21.5%) (80). Although this technology may have limited potential to improve early-stage diagnosis rates in the UK, there may be potential for improvement in other countries, depending on current rates of early detection.

### Strengths and limitations

This systematic review provides a comprehensive summary of novel technologies for GI cancer detection, with findings that are highly relevant to many healthcare systems worldwide. Selection criteria were designed to identify studies that presented high-quality evidence for the technologies under consideration. However, this approach may have resulted in the exclusion of promising technologies that have not yet been evaluated in large, prospective studies. Evidence on the extent to which the identified technologies will increase early-stage diagnosis is lacking. Although modeling studies may offer insights into the potential real-world impact, only prospective evaluations can establish true effectiveness.

### Implications for practice

Technologies used in screening and primary care settings hold the most promise for achieving earlier diagnosis of GI cancers. In secondary care, cancer is likely to have been considered at the time of the referral, and the potential for expedited diagnosis is reduced. Technologies that prompted earlier referral, however, could be considered to expedite the diagnosis. To further the application of any novel technologies in real-world settings, consideration must be given to regulatory requirements, issues around clinical understanding, interpretation of results, and public trust (81,82). Even the highest performing approaches identified by this review, particularly clinical prediction models, lack evidence from external validation in defined target healthcare settings, stakeholder engagement, and health economic evaluation.

### Future research

The CanTest framework (6) provides a conceptual approach for the development and evaluation of cancer diagnostic tests; most approaches identified in this review target stages 2 or 3 of the framework. Future studies should target stages 4 and 5 (evaluation of the test on clinical outcomes such as stage at diagnosis and impact at population level). Promising approaches developed in case-control studies should be validated in external cohort studies to evaluate diagnostic performance in real-world clinical settings.

This review has highlighted novel approaches that could be used to expedite GI cancer diagnosis, including blood-based, stool-based, and algorithm-based strategies. Improvements in diagnostic accuracy may come with challenges in implementation, limiting overall utility. Even relatively simple modifications may provide improvements in diagnostic accuracy (for example, adjusting FIT thresholds). However, whether improvements in diagnostic accuracy translate into meaningful advances in early diagnosis remain uncertain, given the limited evidence specifically relating to early-stage detection. For policy makers considering adoption and implementation of novel technologies, it will be essential to strike the balance between improvements in diagnostic accuracy and timely diagnosis, feasibility, scalability, and cost-effectiveness of widespread use in routine healthcare.

## CONFLICTS OF INTEREST

**Guarantor of the article:** Melissa Barlow, PhD.

**Specific author contributions:** Conception and design: All authors. Study screening and data extraction: M.B., S.E.R.B., S.A., H.A., A.A., P.Z. Data synthesis and analysis: M.B. and S.E.R.B. Data interpretation: All authors. Manuscript preparation: M.B. and S.E.R.B. Manuscript review: all authors.

**Financial support:** This work was supported by the King Faisal Specialist Hospital and Research Center and World Bank. Financing was provided by the King Faisal Specialist Hospital and Research Center and the Health, Nutrition, and Population Reimbursable Advisory Services Programs between the World Bank and the Ministry of Finance in Saudi Arabia (P179873).

**Potential competing interests:** None to report.

## Supplementary Material

## ABBREVIATIONS:

AFP: alpha-fetoprotein
AFP-L3: alpha-fetoprotein-L3
AI: artificial intelligence
AUC: area under the receiver operating characteristic curve
CI: confidence interval
CPM: clinical prediction model
CRC: colorectal cancer
DCP: des-gamma-carboxyprothrombin
FAST: fecal hemoglobin concentration, age, and sex test
FIT: fecal immunochemical test
G-17: gastrin-17
GALADUS: gender, age, alpha-fetoprotein-L3, alpha-fetoprotein, des-gamma-carboxyprothrombin, and ultrasonography
GI: gastrointestinal
H. pylori: Helicobacter pylori
Hb: hemoglobin
HCC: hepatocellular carcinoma
HP-IgG: anti-Helicobacter pylori immunoglobulin G
I^2^: I-squared statistic
MCED: multicancer early detection
NPV: negative predictive value
PG: pepsinogen
PPV: positive predictive value
PRISMA: Preferred Reporting Items for Systematic Reviews and Meta-Analyses
PROSPERO: International Prospective Register of Systematic Reviews
QCancer: cancer risk-prediction algorithm
QUADAS-2: Quality Assessment of Diagnostic Accuracy Studies-2
ROC: receiver operating characteristic
SROC: summary receiver operating characteristic
τ²: between-study variance
UK: United Kingdom
US: ultrasonography
ρ: correlation coefficient
