# Supplementary material for: Novel Technologies for Gastrointestinal Cancer Detection: A Systematic Review and Meta-Analysis of Diagnostic Accuracy
Source: Clin Transl Gastroenterol. 2026 Jun 4;17(8):e01058. doi: 10.14309/ctg.0000000000001058 (PMC13502874; doi:10.14309/ctg.0000000000001058)
Supplement: Supplementary file 1 [file ct9-17-e01058-s003.pdf]

QUADAS-2 Risk of bias assessment for included studies

Colorectal	Risk of bias				Applicability concerns		
Author (year)	Patient selection	Index test	Reference standard	Flow and timing	Patient selection	Index test	Reference standard
Abdullah (2012)	Low	Unclear	Low	High	Low	High	Low
Ayling (2024)	Low	Low	Low	Low	Low	Low	Low
Ball (2022)	Low	Low	Low	Low	Low	Low	Low
Bailey (2021)	Low	Low	Low	Low	Low	Low	Low
Birks 2017	Low	Low	Low	Low	High	Low	Low
Castro (2014)	Low	Low	Unclear	High	Low	Low	Low
Chapman (2020)	Low	Low	Low	Low	Low	Low	Low
Chen (2007)	Unclear	Low	Low	High	Low	Low	Low
Cubiella (2016)	Low	Low	Low	High	Low	Low	Low
Cubiella (2017)	Low	Low	Low	High	Low	Low	Low
D'Souza (2021)	Low	Low	Low	Low	Low	Low	Low
de Wijkerslooth (2012)	Low	Low	Low	Low	Low	Low	Low
Duran-Sanchon (2021)	Low	Unclear	Low	Low	High	Low	Low
Elbeltagi (2021)	Low	Low	Unclear	Low	Low	Low	Low
Erdem (2022)	Low	Low	Low	Low	Low	Low	Low
Farkas (2024)	Low	Low	Low	Low	Low	Low	Low
Guimaraes (2019)	Low	Low	Low	Low	Low	Low	Low
Hernandez (2014)	Low	Low	Low	Low	Low	Low	Low
Herrero (2018)	Low	Low	Low	Low	Low	Low	Low
Högberg (2020)	Low	Low	Low	Low	Low	Low	Low
Hunt (2022)	Low	Low	Low	Low	Low	Low	Low
Imperiale (2014)	Low	Low	Low	Low	Low	Low	Low
Imperiale (2024)	Low	Low	Low	Low	Low	Low	Low
Khan (2020)	Low	Low	Low	Low	Low	Low	High
Khan (2025)	Low	Low	Low	Low	High	Low	Low
Kinar (2016)	Low	Low	Low	Low	Low	Low	Low
Kleif (2022)	Low	Low	Low	High	Low	Low	Low
Li (2024)	Low	Low	Low	Low	Low	Low	Low
Li (2018) (CPM)	Low	Low	Low	High	Low	Low	Low
Li (2018) (FIT)	High	Low	Low	High	High	Low	Low
Luu (2021)	Low	Low	Low	Low	Low	Low	Low
Mahadavan (2012)	Low	Low	Low	Low	Low	Low	Low
Mahadavan (2012) (CPM)	Low	Low	Low	Low	High	Low	Low
Malagon (2023)	Low	Low	Low	Low	Low	Low	Low
MohdSuan (2023)	Low	Low	Low	Low	Low	Low	Low
Nartowt (2019)	Unclear	Low	Low	Low	Unclear	Low	Low
Nicholson (2023)	Low	Low	Low	Low	Low	Low	High
Niedermaier (2017)	Low	Low	Low	Low	Low	Low	Low
Okamura (2020)	Low	High	Unclear	Unclear	Low	Unclear	Unclear
Peterson (2023)	Low	Low	Low	Low	Low	Low	Low
Pin-Vieto (2021)	Low	Low	Low	Low	Low	Low	Low
Rasmussen (2018)	Low	Low	Low	Low	Low	Low	Low

Ross (2022)	Low	Low	Low	Low	Low	Low	Low
Tibbs (2024)	Low	Low	Low	Low	Low	Low	Low
Turvill (2021)	Low	High	Low	Low	Low	Low	Low
Wu (2013)	High	Unclear	Unclear	High	High	Low	Low
Wu, Chen, et al (2022)	Low	Low	Low	Unclear	Low	Low	Low
Wu, Gu, et al (2022)	Low	Low	Low	High	Low	Low	Low
Yen (2014)	Low	Low	Low	Unclear	High	High	Low
Young (2021)	High	Low	Low	High	Low	High	Low
Zhu (2023)	Low	Low	Low	Low	Low	Low	Low

Upper GI

Author (year)	Risk of bias				Applicability concerns		
	Patient selection	Index test	Reference standard	Flow and timing	Patient selection	Index test	Reference standard
Nicholson (2023)	Low	Low	Low	Low	Low	Low	High

Oesophageal

Author (year)	Risk of bias				Applicability concerns		
	Patient selection	Index test	Reference standard	Flow and timing	Patient selection	Index test	Reference standard
Buri (2010)	Low	Unclear	Low	Low	Low	Low	Low

Gastric

Author (year)	Risk of bias				Applicability concerns		
	Patient selection	Index test	Reference standard	Flow and timing	Patient selection	Index test	Reference standard
Cai (2019)	Low	Low	Unclear	Low	Low	Low	Low
Hu (2021)	Unclear	Low	Low	Unclear	Low	Low	Low
Li (2022)	High	High	Low	High	Low	Low	Low
Lomba-Viana (2012)	Low	Low	Low	High	Low	Low	Low
So (2021)	High	High	Low	Low	High	High	Low
Yamamoto (2010)	Low	Low	Low	Low	Low	Low	Low
Zhou (2022)	Low	Unclear	Low	Unclear	High	Low	Low

Pancreatic

Author (year)	Risk of bias				Applicability concerns		
	Patient selection	Index test	Reference standard	Flow and timing	Patient selection	Index test	Reference standard
Boursi (2017)	Low	Low	Low	Low	High	Low	Low
Boursi (2022)	Low	Low	Low	Low	High	Low	Low
Chen (2021)	Low	Low	Low	Low	Low	Low	Low
Chen (2024)	Unclear	Low	Unclear	Low	High	Low	Low
Collins (2013)	Low	Low	Low	Low	Low	Unclear	Low
Hippisley-Cox (2012)	Low	Low	Low	Low	Low	Low	Low
Sharma (2018)	Low	High	Unclear	Unclear	High	Low	Low
Sun (2024)	Low	Low	High	Low	Low	Low	Low
Zhao (2020)	Low	Low	Low	Low	Low	Low	Low

Liver

	Risk of bias				Applicability concerns		

Author (year)	Patient selection	Index test	Reference standard	Flow and timing	Patient selection	Index test	Reference standard
Cao (2023)	Unclear	Low	Low	Unclear	Unclear	Low	Unclear
Chun (2015)	Low	Low	low	Unclear	Unclear	Low	Low
Guo (2024)	Low	Low	Low	Low	Low	Low	Low
He (2025) (GALADUS)	Low	Low	Low	Low	High	Low	Low
He (2025) (AFP + US)	Low	Low	Low	Low	Low	Low	Low
Yu (2023)	Low	Low	Low	Low	Low	Low	Low

QUADAS-2 risk of bias assessment for the included studies.

For studies evaluating more than one test with differing scores, each test is listed separately with an indication of which test the assessment pertains to.
