# Supplementary material for: Novel Technologies for Gastrointestinal Cancer Detection: A Systematic Review and Meta-Analysis of Diagnostic Accuracy
Source: Clin Transl Gastroenterol. 2026 Jun 4;17(8):e01058. doi: 10.14309/ctg.0000000000001058 (PMC13502874; doi:10.14309/ctg.0000000000001058)
Supplement: Supplementary file 2 [file ct9-17-e01058-s001.docx]

**Supplementary materials**

1. Search terms
2. Inclusion and exclusion criteria
3. Summary of included studies
   a. The fecal immunochemical test (FIT) for colorectal cancer detection
   b. Stool-based tests for colorectal cancer detection
   c. Blood-based tests for colorectal cancer detection
   d. Algorithm-based tests for colorectal cancer detection
   e. Other tests for colorectal cancer detection
   f. Tests for upper GI, esophageal, pancreatic, and liver cancer detection
4. Full data extraction table (provided separately in an Excel file)
5. Risk of bias assessment (QUADAS-2) (provided separately as a PDF file)
6. Sensitivity meta-analysis (Asymptomatic FIT ≥20µg Hb/g, excluding Castro (2014)
   a. Forest plot
   b. Summary receiver operator characteristic curve (SROC)
7. Sensitivity meta-analysis (Primary care FIT ≥10µg Hb/g, excluding Farkas (2024)
   a. Forest plot
   b. Summary receiver operator characteristic curve (SROC)

**1. Search terms**

*Ovid MEDLINE and EMBASE*

**Cancer**

1. Cancer.ti,ab.
2. Carcinoma.ti,ab.
3. Adenocarcinoma.ti,ab.
4. 1 or 2 or 3

**Site specific**

5. Colorectal
6. Colon*
7. Rectal
8. Rectum
9. Intestin*
10. Gastr*
11. Stomach
12. Oesophag*
13. Esophag*
14. Liver
15. Hepat*
16. Pancreas
17. Pancreatic
18. 5 or 6 or 7 or 8 or 9 or 10 or 11 or 12 or 13 or 14 or 15 or 16 or 17

**GI cancer specific**

19. 4 adj3 18
20. exp Stomach Neoplasms/
21. exp Colorectal Neoplasms/
22. exp Colonic Neoplasms/
23. exp Intestinal Neoplasms/
24. exp Rectal Neoplasms/
25. exp Esophageal Neoplasms/
26. exp Liver Neoplasms/
27. exp Pancreatic Neoplasms/
28. 19 or 20 or 21 or 22 or 23 or 24 or 25 or 26 or 27

**Diagnosis**

29. exp early detection of cancer/
30. 28 and 29
31. Diagnosis,ti.ab
32. Detection.ti,ab.
33. 31 or 32
34. 33 adj2 28
35. 30 or 34

**Diagnostic performance**

36. Positive predictive value.ti,ab.
37. PPV.ti,ab.
38. 36 or 37
39. Negative predictive value
40. NPV
41. 39 or 40
42. Sensitivity
43. Specificity
44. 38 and 41 and 42 and 43
45. AUC.ti,ab.
46. ROCAUC.ti,ab
47. Area under the curve.ti,ab.
48. Area under the receiver operating characteristic curve.ti,ab.
49. 45 or 46 or 47 or 48
50. 44 or 49
51. 35 and 50

**9372 hits** on Ovid Medline and EMBASE

*Scopus*

TITLE-ABS ((cancer OR carcinoma OR adenocarcinoma) AND (colorectal OR colon OR rectal OR rectum OR intestin* OR gastr* OR stomach* OR oesophag* OR esophag* OR liver OR hepat* OR pancreas OR pancreatic*) AND (diagnos* OR detect*) AND ((positive predictive value OR ppv) AND (negative predictive value OR npv) AND sensitivity AND specificity) OR auc OR rocauc OR area under the curve OR area under the receiver operating characteristic curve)

Limit to: studies published 2005-2025, Medicine field, human, English (no Arabic studies returned)

**3456 studies found**

*Cochrane*

Limit to cancer, diagnosis, and 2005 onwards

**42 reviews returned**

**After deduplication: 9035 studies**

**2. Inclusion and exclusion criteria**

| **Inclusion** | **Exclusion** |
| --- | --- |
| **Participants** | |
| Human adults (≥18 years). | Children (<18 years), cells, animals, tissue samples, images. |
| ≥300 participants . | <300 participants. |
| Patients’ cancer status must be unknown when undergoing testing (to reflect the clinical setting in which the test will be used). | Patients with known cancer at the point of testing. |
| Any country. | No restriction. |
| Any healthcare setting. | Studies that do not explicitly state the healthcare setting in which their participants were recruited. |
| Participants in each study must be from the same healthcare setting (e.g. asymptomatic screening population, primary care, secondary care), unless diagnostic performance metrics are stratified within the study by clinical setting. | Participants from a mixture of clinical settings, and diagnostic performance metrics are not stratified by clinical setting. |
| **Intervention / Exposure** | |
| Test for the *diagnosis* or *detection* of *primary* esophageal, gastric, liver, pancreatic or colorectal cancer. | Tests that do not detect esophageal, gastric, liver, pancreatic or colorectal cancer.  Tests that detect stage, grade, metastasis, or recurrence.  Tests that predict prognosis, treatment response, mortality, or survival.  Tests that monitor tumour progression.  Tests that aim to characterize, distinguish or determine differentiation of tumours, polyps, nodules, masses, lesions. |
| **Comparison** | |
| A diagnosis of esophageal, gastric, liver, pancreatic or colorectal cancer, confirmed via the ‘gold standard’ (e.g. biopsy or electronically thorough the national cancer registry). | A diagnosis of esophageal, gastric, liver, pancreatic or colorectal cancer derived through a non-reference standard test (i.e. comparison with another emerging technology).  A diagnosis of another type of cancer or another condition. |
| **Outcome** | |
| Studies that include sensitivity *and* specificity *and* PPV *and* NPV, *and/or* the AUC for the diagnosis of esophageal, gastric, liver, pancreatic or colorectal cancer *for patients.* | Studies that report only sensitivity (an evaluation of the test only in a cancer population) or specificity (evaluation of the test in a non-cancer population), or measures of artificial intelligence models that do not assess real-world clinical performance.  Diagnostic performance measures for images, nodules, polyps, etc. |
| Tests or technologies designed to improve earlier diagnosis of undiagnosed cancers with a follow up period of <3 years. | Tests or technologies designed to predict future risk of cancer (as opposed to incident cancers), with a follow-up period of ≥3 years. |
| **Types of study** | |
| Cohort study (including non-matched observational studies that may not explicitly state ‘cohort study’) or randomized controlled trials. | Case-control studies, matched observational studies (including matched cohort studies), non-randomized controlled trials. |
| Peer-reviewed, full-texts, theses, pre-prints. | Conference abstracts or studies with no full-text available. |
| Written in English or Arabic. | Written in any other language. |
| Published / written within the last 20 years (2005 – present). | Published before 2005. |

**3a. Summary of included studies evaluating the fecal immunochemical test for colorectal cancer**

| **Author (year)** | **Patients, *n*** | **Incident cancers, *n*** | **Reference standard** | **Positive test threshold, µg Hb/g faeces** | **Sensitivity, % (95% CI)** | **Specificity, % (95% CI)** | **PPV, % (95% CI)** | **NPV, % (95% CI)** | **AUC, % (95% CI)** |
| --- | --- | --- | --- | --- | --- | --- | --- | --- | --- |
| **Asymptomatic / screening** | | | | | | | | | |
| Castro (2014)* | 596 | 6 | Colonoscopy | ≥10 (FIT 1) | 100 (51.7–98.5) | 92.0 (89.5–94.0) | 11.3 (4.7–23.7) | 100 (99.1–100) | FIT 1  0.96 (0.95–0.98)  FIT max  0.95 (0.93–0.97) |
|  |  |  |  | ≥10 (FIT max) | 100 (51.7–98.5) | 89.0 (86.1–91.3) | 8.5 (3.5–18.1) | 100 (99.1–100) |  |
|  |  |  |  | ≥15 (FIT 1) | 100 (51.7–98.5) | 93.6 (91.2–95.3) | 13.6 (5.7–28.1) | 100 (99.1–100) |  |
|  |  |  |  | ≥15 (FIT max) | 100 (51.7–98.5) | 90.7 (88.0–92.8) | 9.8 (4.1–20.9) | 100 (99.1–100) |  |
|  |  |  |  | ≥20 (FIT 1) | 100 (51.7–98.5) | 94.7 (92.5–96.3) | 16.2 (6.8–32.7) | 100 (99.2–100) |  |
|  |  |  |  | ≥20 (FIT max) | 100 (51.7–98.5) | 92.4 (89.8–94.3) | 11.8 (4.9–24.6) | 100 (99.1–100) |  |
|  |  |  |  | ≥23 (FIT 1) | 100 (51.7–98.5) | 95.3 (93.1–96.8) | 17.7 (7.4–35.2) | 100 (99.2–100) |  |
|  |  |  |  | ≥23 (FIT max) | 100 (51.7–98.5) | 93.2 (90.8–95.1) | 13.0 (5.4–27.0) | 100 (99.1–100) |  |
|  |  |  |  | ≥30 (FIT 1) | 83.3 (36.5–99.1) | 95.6 (93.5–97.0) | 16.1 (6.1–34.5) | 99.8 (98.9–100) |  |
|  |  |  |  | ≥30 (FIT max) | 83.3 (36.5–99.1) | 93.7 (91.4–95.5) | 11.9 (4.5–26.4) | 99.8 (98.8–100) |  |
|  |  |  |  | ≥40 (FIT 1) | 66.7 (24.1–94.0) | 95.8 (93.7–97.2) | 13.8 (4.5–32.6) | 99.7 (98.6–99.9) |  |
|  |  |  |  | ≥40 (FIT max) | 66.7 (24.1–94.0) | 94.1 (91.8–95.8) | 10.3 (3.3–25.2) | 99.6 (98.6–99.9) |  |
| de Wijkerslooth (2012) | 1,256 | 8 | Colonoscopy | ≥10 | 88 (47–99) | 91 (89–92) | 6 (3–12) | 100 (99–100) |  |
|  |  |  |  | ≥15 | 75 (36–96) | 93 (92–95) | 7 (3–15) | 100 (99–100) |  |
|  |  |  |  | ≥20 | 75 (36–96) | 95 (93–96) | 8 (3–18) | 100 (99–100) |  |
| Hernandez (2014) | 779 | 5 | Colonoscopy | ≥10 | 100 (90–100) | 92 (90–94) | 7 (0–15) | 100 (100–100) | 0.97 (0.94–0.99) |
|  |  |  |  | ≥15 | 100 (90–100) | 93 (91–95) | 8 (0–16) | 100 (100–100) |  |
|  |  |  |  | ≥20 | 100 (90–100) | 94 (92–95) | 9 (1–18) | 100 (100–100) |  |
|  |  |  |  | ≥23 | 100 (90–100) | 94 (92–96) | 10 (1–19) | 100 (100–100) |  |
|  |  |  |  | ≥30 | 80 (35–100) | 94 (93–96) | 8 (0–17) | 100 (100–100) |  |
|  |  |  |  | ≥40 | 80 (35–100) | 95 (93–96) | 9 (0–18) | 100 (100–100) |  |
| Li (2018) | 55,038 | 311 | Colonoscopy | Presence of Hb |  |  |  |  | 0.76 (0.74–0.79) |
| Malagon (2023) | 2,481 | 15 | Colonoscopy | ≥20 | 66.7 | 96.1 | 9.5 | 99.8 |  |
| Niedermaier (2017) | 3,466 | 30 | Colonoscopy |  |  |  |  |  | 0.96 (0.91–0.99) |
| **Primary care** | | | | | | | | | |
| Ayling (2024) | 14,428 | 122 | Colonoscopy | ≥10 | 95.1 (89.6–98.2) | 77.7 (77.0–78.4) | 3.5 (2.9–4.2) | 99.95 (99.9–99.98) |  |
| Bailey (2021) | 3890 | 51 | Medical records | ≥10 | 84.3 (71.4–93.0) | 85.0 (83.8–86.1) | 7.0 (5.1–9.3) | 99.8 (99.5–99.9) | 0.92 (0.86–0.96) |
| Ball (2022) | 3506 | 45 |  | ≥10 | 91.1 (77.9–97.1) | 80.7 (79.3–82.0) | 5.8 (4.2–7.8) | 99.9 (99.6–99.95) | 0.93 (0.91–0.96) |
|  |  |  |  | ≥20 | 86.7 (72.5–94.5) | 87.3 (86.1–88.4) | 8.1 (5.9–11.1) | 99.8 (99.5–99.9) |  |
|  |  |  |  | ≥50 | 73.3 (57.8–84.9) | 93.0 (92.0–93.8) | 11.9 (8.4–16.4) | 99.6 (99.3–99.8) |  |
|  |  |  | Colonoscopy | ≥80 | 66.7 (50.9–79.6) | 94.9 (94.2–95.7) | 14.7 (10.3–20.4) | 99.5 (99.2–99.7) |  |
|  |  |  |  | ≥100 | 66.7 (50.9–79.6) | 95.8 (95.0–96.4) | 17.0 (12.0–23.6) | 99.5 (99.2–99.7) |  |
|  |  |  |  | ≥120 | 62.2 (46.5–75.8) | 96.2 (95.5–96.8) | 17.6 (12.2–24.6) | 99.5 (99.1–99.6) |  |
|  |  |  |  | ≥150 | 53.3 (38.0–68.1) | 96.9 (96.3–97.4) | 18.3 (12.3–26.2) | 99.4 (99.0–99.6) |  |
| Chapman (2020) | 1680 | 36 | Colonoscopy | ≥150 | 69.4 (51.9–83.6) | 93.5 (91.7–95.1) | 30.9 (24.2–38.4) | 98.7 (97.8–98.2) |  |
|  |  |  |  | <4.0 | 97.2 (85.5–99.9) | 61.4 (58.1–64.7) | 9.5 (8.7–10.4) | 99.8 (98.7–100.0) |  |
| D'Souza (2021) | 9822 | 329 (3.3%) | Colonoscopy | ≥2 | 97.0 (94.5–98.5) | 64.9 (63.9–65.8) | 8.7 (7.8–9.7) | 99.8 (99.7–99.9) | 0.93 (0.92–0.95) |
|  |  |  |  | ≥10 | 90.9 (87.2–93.8) | 83.5 (82.8–84.3) | 16.1 (14.4–17.8) | 99.6 (99.5–99.7) |  |
|  |  |  |  | ≥150 | 70.8 (65.6–75.7) | 94.6 (94.1–95.0) | 31.1 (27.8–34.6) | 98.9 (98.7–99.1) |  |
| Farkas (2024) | 460 | 23 | Colonoscopy | ≥10 (1 x FIT) | 95.7 (78.1–99.9) | 44.6 (39.9–49.4) | 8.3 (7.5–9.3) | 99.5 (96.6–99.9) |  |
|  |  |  |  | ≥10 (2 X FIT) | 95.7 (78.1–99.9) | 40.7 (36.1–45.5) | 7.8 (7.0–8.7) | 99.4 (96.3–99.9) |  |
|  |  |  |  | ≥10 (3 x FIT) | 95.7 (78.1–99.9) | 38.4 (33.9–43.2) | 7.6 (6.8–8.4) | 99.4 (96.1–99.9) |  |
| Högberg (2020) | 5683 | 107 | Cancer registry | 25–50 | 95.3 | 68.5 | 5.5 (4.5–6.5) | 99.9 (99.8–100) |  |
| Pin-Vieto (2021) | 54327 | 650 | Medical records | ≥10 | 90.5 (99.0–92.5) | 81.4 (81.0–81.8) | 7.7 (7.1–8.3) | 99.8 (99.7–99.8) | 0.89 (0.88–0.91) |
|  |  |  |  | ≥20 | 87.7 (84.9–90.0) | 86.7 (86.3–87.0) | 10.1 (9.3–10.9) | 99.8 (99.7–99.8) |  |
| Tibbs (2024) | 3119 | 12 | Colonoscopy | ≥10 | 100 (75.8–100) | 86.3 (85.1–87.5) | 2.7 (2.5–3.0) | 100 | 0.89 (0.82–0.85) |
|  |  |  |  | ≥150 | 83.3 (55.2–95.3) | 92.5 (94.4–95.9) | 6.2 (4.7–8.2) | 99.9 (99.8–100) |  |
| **Secondary care** | | | | | | | | | |
| Elbeltagi (2021) | 992 | 52 | Colonoscopy | ≥7 | 94.2 | 75.3 | 17.4 | 99.6 | 0.89 (0.85–0.93) |
| Guimares (2019) | 368 | 37 | Colonoscopy | Presence of Hb (3 x FIT) | 91.9 (78.1–98.3) | 68.0 (60.5–74.9) | 38.2 (32.8–44.0) | 97.5 (92.9–99.1) | 0.80 (0.73–0.87) |
|  |  |  |  | Presence of Hb/Hp (3 X FIT) | 94.6 (81.8–99.3) | 65.1 (57.5–72.2) | 36.8 (31.9–42.1) | 98.2 (93.5–99.5) | 0.80 (0.73–0.87) |
| Herrero (2018) | 1572 | 214 | Colonoscopy | ≥10 | 93.5 (89.1–96.3) | 63.4 (60.7–66.0) | 28.9 (25.6–32.4 | 98.4 (97.2–99.1) | 0.86 (0.84–0-89) |
|  |  |  |  | ≥20 | 98.4 (97.2–99.1) | 69.6 (67.1–72.0) | 32.3 (28.6–36.2) | 98.0 (96.9–98.8) |  |
| Hunt (2022) | 28,622 | 319 | Cancer registry | ≥10 (2 x FIT, either positive) | 97.8 (95.5–98.9) | 66.2 (65.7–66.7) | 3.1 (2.8–3.5) | 100 (99.9–100) |  |
|  |  |  |  | ≥10 (2 x FIT, both positive) | 91.5 (87.0–94.1) | 81.6 (81.1 to 82.0) | 5.2 (4.6–5.8) | 99.9 (99.8–99.9) |  |
| Khan (2020) | 928 | 47 | Cancer registry | ≥10 | 85.1 (71.0–93.3) | 83.5 (83.8–85.8) | 22.6 (16.0–28.3) | 99.0 (97.9–99.5) | 0.89 (0.84–0.94) |
| Turvill (2018) | 505 | 27 | Cancer registry | ≥12 | 84.6 | 88.5 | 28.6 | 99.1 | 0.87 (0.79–0.94) |
|  |  |  |  | ≥43 (2 x FIT, either positive) | 87.5 | 90.7 | 33.3 | 99.3 | 0.89 (0.82–0.96) |
|  |  |  |  | ≥2 (2 x FIT, both positive) | 91.7 | 85.2 | 24.7 | 99.5 | 0.84 (0.83–0.94) |
| Turvill (2021) | 5040 | 151 | Colonoscopy or CT colonography or a lesser investigation (e.g. CT abdomen / pelvis with contrast or flexible sigmoidoscopy | ≥19 | 85.4 (78.8–90.6) | 85.2 (84.1–86.2) | 15.1 (12.8–17.7) | 99.5 (99.2–99.7) | 0.89 (0.86–0.92) |

**Table X: Summary of included studies that reported the diagnostic accuracy of FIT for CRC detection.**

Thresholds were converted from ng/mL to µg Hb/g feces for comparability.

The sensitivity, specificity, PPV and NPV for Ayling (2024) has been manually calculated to combine their two cohorts that were separated by year of FIT (2020 and 2021).

* Castro’s patients were asymptomatic, but high risk in that they had at least one first-degree relative who has / has had colorectal cancer

FIT fecal immunochemical test; FIT1 patient’s first FIT; FIT max patient’s maximum FIT.

**3b. Summary of included studies evaluating stool-based tests for colorectal cancer**

| **Author (year)** | **Test / technology** | **Patients, *n*** | **Incident cancers, *n*** | **Reference standard** | **Sensitivity, % (95% CI)** | **Specificity, % (95% CI)** | **PPV, % (95% CI)** | **NPV, % (95% CI)** | **AUC, % (95% CI)** |
| --- | --- | --- | --- | --- | --- | --- | --- | --- | --- |
| **Asymptomatic** | | | | | | | | | |
| Chen (2007) | FOBT (≥100ng/mL) | 22,672 | 65 | Colonoscopy or cancer registry | 81.5 (70.2–89.2) | 94.3 (94.0–94.6) |  |  | 0.87 (0.81–0.93) |
| Impariale (2014) | Multitarget Stool DNA | 9,989 | 65 | Colonoscopy | 92.3 (83.0–97.5) |  | 3.7 (2.9–4.8) | 99.9 (99.8–1.00) | 0.94 |
| Imperiale (2024) | Next-generation multitarget stool DNA test | 20,176 | 98 | Colonoscopy | 93.9 (87.1–97.7) | 87.0* | 3.4 (2.7–4.1) | 99.97 (99.9–99.99) | 0.98 (0.96–1.00) |
| Malagon (2023) | FIT + fecal bacteria | 2481 | 15 | Colonoscopy | 66.7 | 97 | 11.9 | 99.8 |  |
| Ross (2022) | FC ≥50µg/g) | 352 | 12 | Colonoscopy | 92.8 (66.1–99.8) | 41.7 (36.4–47.2) | 6.2 (5.3–7.3) | 99.3 (95.5–99.9) |  |
|  | FC ≥200µg/g |  |  |  | 64.3 (35.1–87.2) | 74.9 (69.9–79.4) | 9.6 (6.4–14.0) | 98.1 (96.2–99.0) |  |
| Wu (2013) | iFOBT | 1481 | 6 | Colonoscopy | 83.3 | 87.2 | 2.8 | 99.9 |  |
| Young (2021) | FOBT + microbiome | 2,252 | 430 | Colonoscopy |  |  |  |  | 0.86 (0.82–0.89) |
| Zhu (2023) | Stool DNA | 4,293 | 8 | Colonoscopy | 100 (60–100) | 84 (82–85) |  |  | 0.96 (0.95-0.97) |
| **Secondary care** | | | | | | | | | |
| Abdullah (2012) | M2PK | 328 | 42 | Colonoscopy | 71.40% | 71 | 73.5 | 94.4 |  |
| Guimaraes (2019) | gFOBT | 368 | 37 | Colonoscopy | 75.7 (58.8–88.2) | 84.3 (78.0–89.4) | 50.9 (41.2–60.5) | 94.2 (90.1–96.6) | 0.80 (0.71–0.89) |
| MohdSuan (2023) | M2PK | 562 | 17 | Colonoscopy | 58.8 (32.9–81.6) | 85.5 (82.3–88.4) | 11.2 (7.5–16.5) | 98.5 (97.4–99.2) |  |
| Turvill (2018) | FC ≥118µg/g | 510 | 27 | Colonoscopy or CT colonography or a lesser investigation (e.g. CT abdomen / pelvis with contrast or flexible sigmoidoscopy | 74.1 | 66.3 | 10.9 | 97.9 | 0.70 (0.62–0.79) |
|  | 2 x FC ≥151µg/g (either positive) | 481 | 27 |  | 76 | 65.4 | 10.7 | 98 | 0.71 (0.62–0.80) |
|  | 2 x FC ≥78µg/g (both positive) | 481 | 27 |  | 68 | 65.4 | 9.7 | 97.4 | 0.67 (0.57–0.76) |
|  | 2 x FIT ≥2µgHb/g + 2 x FC ≥10µg | 474 | 27 |  | 91.7 | 85.8 | 25.6 | 99.5 | 0.89 (0.83–0.95) |
|  | 2 x FIT ≥2µgHb/g + 1 x FC ≥10µg | 474 | 27 |  | 91.7 | 85.3 | 25 | 99.5 | 0.89 (0.83–0.94) |
|  | 2 X FIT ≥2µgHb/g + 2 x FC ≥10µg | 474 | 27 |  | 91.7 | 85.1 | 24.7 | 99.5 | 0.88 (0.83–0.94) |
|  | 2 X FIT ≥2µgHb/g + 2 x FC ≥20µg | 474 | 27 |  | 87.5 | 86.7 | 25.9 | 99.2 | 0.87 (0.80–0.94) |
|  | 1 x FIT ≥2µgHb/g + 1 x FC ≥10µg | 503 | 27 |  | 84.6 | 88.7 | 28.9 | 99.1 | 0.87 (0.79–0.94) |

FC fecal calprotectin; FIT fecal immunochemical test, FOBT fecal occult blood test, gFOBT guaiac-based fecal occult blood test; Hb hemoglobin; iFOBT immunochemical fecal occult blood test; M2PK Tumor M2-Pyruvate Kinase

* Manually calculated statistic from raw figures

**3c. Summary of included studies for blood-based tests for colorectal cancer**

| **Author (year)** | **Test / technology** | **Patients, *n*** | **Incident cancers, *n*** | **Reference standard** | **Sensitivity, % (95% CI)** | **Specificity, % (95% CI)** | **PPV, % (95% CI)** | **NPV, % (95% CI)** | **AUC, % (95% CI)** |
| --- | --- | --- | --- | --- | --- | --- | --- | --- | --- |
| Asymptomatic | | | | | | | | | |
| Okamura (2020) | Triglyceride glucose index | 27921 | 116 | Unclear | 62 | 66.8 |  |  | 0.69 (0.64-0.74) |
| Secondary care | | | | | | | | | |
| Nicholson (2023) | MCED | 2202 | 143 | Clinical diagnosis | 68.5 | 99.1 | 83.8 | 97.8 |  |
| Rasmussen (2018) | Cell-free nucleosomes | 4105 | 441 | Colonoscopy |  |  |  |  | 0.74 |

MCED multi cancer early detection

**3d. Summary of included studies for algorithm-based tests for colorectal cancer**

| **Author (year)** | **Model** | **Variables in model** | **Patients, *n*** | **Incident cancers, *n*** | **Reference standard** | **Sensitivity, % (95% CI)** | **Specificity, % (95% CI)** | **PPV, % (95% CI)** | **NPV, % (95% CI)** | **AUC, % (95% CI)** |
| --- | --- | --- | --- | --- | --- | --- | --- | --- | --- | --- |
| Asymptomatic | | | | | | | | | | |
| Erdem (2022) | CPM | Age, sex, BMI, smoking, alcohol consumption, family history | 6,508 | 150 | Colonoscopy | 96% | 39% | 3.4 (3.1-3.6) | 99.3 (98.6-99.6) | 0.71 (0.66-0.76) |
| Kleif (2022) | CPM (FIT+) | Age, sex, and blood markers (CEA, ferritin, hsCRP, HE4) | 4,048 | 242 | Cancer registry |  |  |  |  | 0.70 (0.66-0.74) |
| Li (2018) | CPM | Age, sex, socioeconomic factors, clinical diagnoses history, family history | 891,199 | 471 | Colonoscopy |  |  |  |  | 0.73 (0.71-0.76) |
|  | CPM | The above + FIT | 550,318 | 311 |  |  |  |  |  | 0.84 (0.82-0.86) |
| Li (2024) | Multiple AI models and CPM | Age, sex, FOBT, socioeconomic factors, lower GI symptoms, rurality, cancer history, polyps, family history, clinical diagnoses, trauma | 359462 | 728 | Colonoscopy |  |  |  |  | Ranging from 0.37–0.86 |
| Luu (2021) | CPM (APCS) | Age, sex, family history, smoking status | 12,520 | 110 | Cancer registry |  |  |  |  | 0.62 |
| Nartowt (2019) | AI model | Age, sex BMI, smoking status, clinical diagnoses, family history, exercise frequency, ethnicity | 58,376 | 140 | Clinical diagnosis | 57 ± 3 | 89 ± 2 | 0.75 ± 0.03 | 99.9 ± 0.1 | 0.80 ± 0.05 |
| Petersen (2023) | CPM (FIT+) | FIT result, age, CEA, hsCRP and Ferritin | 4,048 | 242 | Colonoscopy |  |  |  |  | 0.69 (0.66-0.72) |
|  | CPM (FIT+) | FIT result, age, sex, CEA, hsCRP, TIMP, Pepsinogen-2, HE4, CyFra21-1, galectin-3, ferritin, B2M |  |  |  |  |  |  |  | 0.74 (0.66-0.82) |
| Wu, Chen, et al (2022) | CPM (FIT+) | Age, sex, rurality, lower GI symptoms, and family history | 140,475 | NS | Colonoscopy |  |  |  |  | 0.63 (0.60–0.66) |
|  | CPM (FIT-) | As above + diarrhea and previous cancer | 20,951 |  |  |  |  |  |  | 0.64 (0.58–0.69) |
|  | CPM | As above + FIT | 161,426 |  |  |  |  |  |  | 0.86 (0.84–0.87) |
|  | AI model (FIT +) | Unclear | 140,475 |  |  |  |  |  |  | 0.63 (0.60–0.66) |
|  | AI model (FIT -) |  | 20,951 |  |  |  |  |  |  | 0.63 (0.58–0.68) |
|  | AI model |  | 161,426 |  |  |  |  |  |  | 0.85 (0.84–0.87) |
| Wu, Gu, et al (2022) | CPM | Age, sex, family history, lower GI symptoms, cancer history | 322,880 | NS | Colonoscopy |  |  |  |  | 0.65 (0.63–0.66) |
|  | AI model | As above |  |  |  |  |  |  |  | 0.65 (0.63–0.66) |
|  | CPM | As above + 1 x FIT |  |  |  |  |  |  |  | 0.79 (0.77–0.80) |
|  | AI model | As above |  |  |  |  |  |  |  | 0.79 (0.77–0.80) |
|  | CPM | As above + 2 x FIT |  |  |  |  |  |  |  | 0.81 (0.80–0.83) |
|  | AI model | As above + 2 x FIT |  |  |  |  |  |  |  | 0.81 (0.80–0.83) |
| Yen (2014) | CPM | fHB, sex, family history, clinical diagnoses, alcohol, smoking, BMI | 54,921 | 323 | Colonoscopy or cancer registry |  |  |  |  | 0.79 (0.77–0.81) |
| Primary care | | | | | | | | | | |
| Birks 2017 | AI model | Age, sex, and full blood count measure | 2,225,249 | 5141 | Colonoscopy |  |  |  |  | 0.78 (0.77–0.79) |
| Kinar (2016) | AI model | Age, sex, and full blood count measure | 139,205 | 698 | Cancer registry |  |  |  |  | 0.82 +- 0.01 |
| Secondary care | | | | | | | | | | |
| Cubiella 2016 | CPM  (COLONPREDICT, ≥3.5) | f-Hb, age, sex, lower GI symptoms, serum CEA, Hb, aspirin, previous colonoscopy | 1481 | 136 | Colonoscopy | 100 (96.0–100) | 46.8 (44.0–49.6) | 3.5 (2.1–5.9) |  | 0.92 (0.90 –0.94) |
|  | CPM  (COLONPREDICT, ≥5.6) |  |  |  |  | 87.1 (79.9–92.1) | 79.3 (76.9–81.4) | 30.3 (25.8-35.3) |  |  |
| Cubiella 2017 | CPM (FAST, ≥2.1) | f-Hb, age, and sex | 3,976 | 207 | Colonoscopy | 100 (97.8–100) | 13.9 (12.1–15.9) | 15.6 (13.7–17.6) | 100 (97.5–100) | 0.91 (0.90–0.93) |
|  | CPM (FAST, ≥4.5) |  |  |  |  | 89.8 (84.7 –93.3) | 71.3 (68.8–73.7) | 33.2 (29.4–37.2) | 97.8 (96.6–98.6) |  |
| Duran-Sanchon 2021 | CPM | f-Hb, age, sex, miR-421, miR-27a-3p | 767 | 67 | Colonoscopy |  |  |  |  | 0. 89.9 (85.8–93.7) |
| Mahadavan (2012) | CPM | Age, sex, FOBT, rectal bleeding, mean red blood cell volume, DNA from colonocytes | 714 | 72 | Biopsy |  |  |  |  | 0.88 (0.84-0.92) |

AI artificial intelligence; APCS Asia Pacific Colorectal Screening score; B2m beta-2 microglobulin; BMI body mass index; CEA carcinoembryonic antigen; CPM clinical prediction model; CyFra21-1 fragment of cytokeratin 19; f-Hb fecal hemoglobin; FC fecal calprotectin; FIT fecal immunochemical test; FOBT fecal occult blood test; GI gastrointestinal; HE4 Human Epididymis Protein 4; hsCRP high sensitivity c-reactive protein; miR micro RNA; TIMP Tissue Inhibitors of Metallopriteinases.

(FIT+) or (FIT-) indicates FIT positive or FIT negative population.

* High risk population (from FIT result, questionnaire)

**3d. Summary of included studies for other tests for colorectal cancer**

| **Author (year)** | **Test / technology** | **Healthcare setting** | **Patients, *n*** | **Incident cancers, *n*** | **Reference standard** | **Sensitivity, % (95% CI)** | **Specificity, % (95% CI)** | **PPV, % (95% CI)** | **NPV, % (95% CI)** | **AUC, % (95% CI)** |
| --- | --- | --- | --- | --- | --- | --- | --- | --- | --- | --- |
| Mahadavan (2012) | Exfoliated colonocyte DNA | Secondary care | 714 | 72 | Biopsy |  |  |  |  | 0.65 (0.58-0.72) |
| Niedermaier (2017) | Flexible sigmoidoscopy + FIT ≥27µg Hb/g | Screening | 3466 | 29 | Colonoscopy | 95 | 100 (88–100) |  |  | 99.6 (0.99–1.00) |

FIT fecal immunochemical test; Hb hemoglobin

**3e. Summary table for upper GI, esophageal, pancreas, and liver**

| **Author (year)** | **Test / technology** | **Healthcare setting** | **Patients, *n*** | **Incident cancers, *n*** | **Reference standard** | **Sensitivity, % (95% CI)** | **Specificity, % (95% CI)** | **PPV, % (95% CI)** | **NPV, % (95% CI)** | **AUC, % (95% CI)** |
| --- | --- | --- | --- | --- | --- | --- | --- | --- | --- | --- |
| **Upper GI** | | | | | | | | | | |
| Nicholson (2023) | MCED | Secondary care | 1021 | 46 | Upper endoscopy | 80.4 | 98.1 | 66.1 | 99.1 |  |
| **Esophageal** | | | | | | | | | | |
| Buri (2010) | CPM | Secondary care | 8,252 | 215 | Upper endoscopy | 60 (53–67) | 89 (88–90) | 9 (7–11) | 99 (99–99.4) | 0.82 (0.78–0.86) |
|  | AI model | Secondary care | 8,252 | 215 |  | 83 (77–90) | 82 (81–83) | 7 (6–9) | 99.6 (99.3–99.8) | 0.87 (0.83–0.89) |
| **Gastric** | | | | | | | | | | |
| Cai (2019) | CPM (including PGs and Hp) | Asymptomatic | NR | 138 | Gastroscopy and biopsy | 69.6 | 66.8 | 5.5 | 98.7 | 0.76 (0.73–0.79) |
| Hu (2021) | CPM (ABC) | Asymptomatic | 6005 | 72 | Gastroscopy and biopsy | 25 | 85 |  |  | 0.57 (0.50–0.64) |
|  | CPM (New ABC) |  |  |  |  | 25 | 85 |  |  | 0.50 (0.42–0.57) |
|  | CPM (Tu) |  |  |  |  | 94 | 15 |  |  | 0.53 (0.46–0.61) |
|  | CPM (Li) |  |  |  |  | 75 | 57 |  |  | 0.71 (0.64–0.77) |
| Li (2022) | Serum PGI, PGII, PGR, G-17, Hp | Targeted screening* | 1019 | 24 | Gastroscopy and biopsy | 81.5 | 77.8 |  |  | 0.82 (0.72–0.91) |
| Lomba-Viana (2012) | Serum PGI and PGII | Asymptomatic | 13118 | 6 | Gastroscopy and biopsy | 67 (63 –71) | 47 (43 –51) | 2 (1 –3) | 99 (98 –100) |  |
| So (2021) | 12-miR | Secondary care | 4566 | 125 | Gastroscopy and biopsy |  |  |  |  | 0.85 (0.81–0.88) |
| Yamamoto (2010) | Radiography (high-density barium sulphate) | Asymptomatic | 48336 | 73 | Cancer registry | 91.8 | 91.4 | 1.6 |  | 0.94 |
|  | Radiography (medium density barium sulphate) |  | 123497 | 246 |  | 92.3 | 91 | 2 |  | 0.93 |
| Zhou (2022) | Clinical scoring system | Secondary care | 6701 | 190 | Gastroscopy and biopsy |  |  |  |  | 0.79 |
| **Pancreas** | | | | | | | | | | |
| Boursi (2017) | CPM | New onset diabetes | 109,385 | 390 | Medical records | 44.7 | 94.0 | 2.6 |  | 0.82 (0.75–0.89) |
| Boursi (2022) | CPM | Pre-diabetes | 138,232 | 245 | Medical records | 9.3 | 99.7 | 4.7 |  | 0.71 (0.67–0.75) |
| Chen (2021) | CPM (END-PAC) | Elevated HbA1c or abnormal glucose | 13,947 | 99 | Cancer registry | 62.6 | 78.5 | 2.0 | 99.7 | 0.75 |
| Chen (2024) | CPM (modified END-PAC) | Elevated HbA1c | 69,334 | 329 | Cancer registry |  |  |  |  | 0.75 |
| Collins (2013) | CPM (Q-cancer) | Primary care | 2150322 | 618 | Medical records | 63.8 | 99 | 0.2 | 100 | 0.89 males  0.92 females |
| Hippisley-Cox (2012) | CPM (Q-cancer) | Primary care | 1243740 | 781 | Medical records |  |  |  |  | 0.87 (0.85–0.88) males  0.84 (0.82–0.86) females |
| Sharma (2018) | CPM (END-PAC) | New-onset diabetes | 1096 | 9 | Unclear |  |  |  |  | 0.87 |
| Sun (2024) | CPM | Type 2 diabetes | 12735 | 100 | Cancer registry |  |  |  |  | 0.71 (0.66–0.76) |
|  | CPM + genetics | Type 2 diabetes | 12735 | 100 |  | 77 | 83.7 | 3.6 | 99.8 | 0.90 (0.87–0.93) |
|  | Genetics prediction model | Type 2 diabetes | 12735 | 100 |  |  |  |  |  | 0.88 (0.84–0.92) |
| Zhao (2020) | CPM | Chronic pancreatitis | 415 | 7 | Medical records | 0.71 (0.43–1.00) | 0.85 (0.45–0.94) |  |  | 0.73 (0.48–0.97) |
| **Liver** | | | | | | | | | | |
| Cao (2023) | AFP (≥20ng/mL) | Hepatitis B | 1205 | 39 | Medical records |  |  |  |  | 67.57 (57.26–77.88) |
|  | US |  |  |  |  |  |  |  |  | 83.26 (74.71–91.82) |
|  | AFP (≥20ng/mL) + US |  |  |  |  |  |  |  |  | 86.45 (79.56–93.52) |
|  | AFP+US+ctDNA (≥15ng/mL) |  |  |  |  | 84.89 | 97.7 |  |  | 0.93 (0. 90–0.97) |
|  | AFP+US+ctDNA (≥20ng/mL) |  |  |  |  | 91.7 | 97.6 |  |  |  |
|  | AFP+US+ctDNA (≥25ng/mL) |  |  |  |  | 100 | 97.3 |  |  |  |
| Chun (2015) | AFP (≥8.1ng/mL) | Asymptomatic | 48,122 | 24 | Medical records | 82.3 | 99.99 | 1.7 | 99.99 | 0.96 (0.91–1.0) |
| Guo (2024) | ALARM model (≥0.21) | Cirrhosis | 703 | 31 | Histology or radiology | 94.4 (95.5–98.1) | 68.4 (66.2 to 73.0) | 13.0 (10.3–15.3) | 99.6 (99.4–99.9) | 0.92 (0.90–0.96) |
|  | ALARM model (≥0.65) |  |  |  |  | 64.5 (61.0–68.1) | 92.6 (90.6 to 94.5) | 28.6 (25.2–31.9) | 98.3 (97.3–99.2) |  |
| Yu (2023) | CPM (M2P-HCC) | Hepatitis B | 313 | 41 | Liver US, enhancement, CT enhancement, and/or tumor-specific MRI |  |  |  |  | 0.88 (0.81–0.95) |
|  | AFP + US |  |  |  |  |  |  |  |  | 0.76 (0.69–0.84) |

**Table X: Summary of extracted studies reporting diagnostic performance measures for tests and technologies for the detection of upper GI, oesophageal, gastric, pancreatic and liver cancers.**

AFP alpha-fetoprotein; AI artificial intelligence; ALARM Algorithm for Early Warning of Hepatocellular Carcinoma; CPM clinical prediction model; ctDNA circulating tumor DNA; END-pac Enriching New-onset Diabetes for Pancreatic Cancer; M2P-HCC (Methylation, Mutation, and Protein- Hepatocellular Carcinoma); MCED multi cancer early detection; PG serum pepsinogen; US ultrasound

* Medium / high risk, based on blood test and questionnaire

6. **Sensitivity meta-analysis (Asymptomatic FIT, excluding Castro (2014)**
   a. **Forest plot**

b. **SROC**

7. **Sensitivity meta-analysis (FIT in primary care ≥10μg Hb / g, excluding Farkas (2024)**
a. **Forest plot**

b. **SROC**
